# Principals' leadership styles and its impact on teachers' performance at college level

**DOI:** 10.3389/fpsyg.2022.919693

**Published:** 2022-09-06

**Authors:** Uzma Sarwar, Rameez Tariq, Qi Zhan Yong

**Affiliations:** ^1^School of Education, Shaanxi Normal University, Xi'an, China; ^2^School of Education, Huanggang Normal University, Huanggang, China; ^3^Management Sciences Department, COMSATS University Islamabad, Islamabad, Pakistan

**Keywords:** principal, leadership styles, teachers performance, college, Pakistan

## Abstract

In this study, we examined the impact of principals' leadership style on the performance of teachers at the college level. For this purpose, we collect data from 300 college teachers *via* a random sampling approach. A self-administrated questionnaire (five-point Likert Scale) was used to collect data. For detecting relationships and differences among the opinions of the study's participants, correlation and the *t*-test were used. This study has revealed that the majority of college principals practice a democratic style of leadership at a higher level, Laissez-faire at a moderate level, and autocratic at a low level in their colleges. Moreover, it has been also revealed that when principals increase the use of a democratic leadership style, teacher performance may progress as well. The findings revealed that principals' leadership style had a positive impact on the performance of teachers. The study exposed a strong statistically positive relationship between college principals' leadership style and teacher performance. Thus, the results of this study suggest that college principals' should adopt the leadership style according to the level of teachers. The leadership style should be changed with specific situations in the colleges.

## Introduction

Effective leadership is necessary for the advancement of teachers as well as society. In the technological advancement of the 21st century, there are many challenges to compete including worldwide teachers' networks which demand a great educational leader for educational institutions. There are three main aspects of a principal's leadership in dealing with educational and cultural reforms such as increasing participation, transferring vision, and producing change. The effectiveness of leaders in the educational sector is valued by their competencies to contribute to improving the quality of education in the era of technological advancement (Sungtong, 2007; Abbas et al., 2020).

The main job of a principal is to assist in leading, directing, and coordinating various activities inside the college. The primary responsibility of the principal is to create and sustain an excellent teaching-learning environment for the educational programs running in the college. The principal is also responsible to support the teachers in their teaching practices. Principals have a critical role to play in achieving the institution's goals and objectives. Among these responsibilities, principals must give genuine and effective leadership, resulting in improved professional presentation among teachers. The principal is responsible to give highly valued visions that are focused on their day-to-day methods and that serve to foster a good culture that is supportive of exceptional teacher performance (Nanson, 2010; Saleem et al., 2020).

In various locations and circumstances, a lot of researchers explained leadership such as Okumbe (1998) describes leadership as “a specific attitude adopted by a leader toward his or her subordinates to motivate them to achieve the organization's objectives and goals.” Western (2013) states that leadership is recognized as the abilities and practical skills of the persons, groups, or organizations to lead, influence, or provide guidance to other persons, teams, or the whole organization. Chin (2015) explains leadership in the context of the American academic environment as a process of social influence through which an individual can enlist the aid and support of others in the attainment of common as well as ethical tasks. Northouse (2018) and Wu et al. (2020) highlight that leadership is a prominent power relationship in which one party (leader) promotes movements or changes in others (followers).

Furthermore, Phuc et al. (2021) also found that a leadership style refers to a leader's style of giving directions, implementing plans, and motivating followers. Among others Bhoomireddy (2004); Goel (2005), and Crum and Sherman (2008) a leader uses a different style of leadership considering the situation. In situations of emergency, an autocratic style of leadership is considered more effective while for a highly motivated and aligned team democratic or laissez-faire styles are recognized as more effective (Department of the Army, 2006).

## Problem statement

Besides, this study is proposed to measure the impact of different principal leadership styles on the teacher's performance currently serving in colleges in the major with the highest rate of population province Punjab, Pakistan. Although wide research has been conducted to report this phenomenon from different perspectives and in different contexts, leftovers anonymous has not yet adequately resulted. The linkage between principal leadership styles and teacher performance is still mostly unmapped in the context of Pakistan (Quraishi and Aziz, 2018; Maqbool et al., 2019; Yasmin et al., 2019; Saleem et al., 2020). Especially, the govt. college teachers in many Pakistani cities for example Faisalabad, Punjab. Therefore, concerning education in Pakistan, more clarification is imperative to determine how and what style of leadership for principals positively or negatively affects teacher performance at the college level in Faisalabad.

## Research objectives

These objectives were developed for this study:

1. To explore the leadership styles of principals practiced at the college level.
2. To examine the relationship between a principal's leadership style and the performance of teachers at the college level.

## Research questions

1. Whether the style of leadership matter in the case of a college principal?
2. Whether a principal's leadership style effects the performance of teachers at the college level?

## Hypotheses

Based on prior studies and extant literature review, the following are the hypotheses of the study:

**H1:** There is a significant role of principal leadership style at the college level.

**H2:** There is a significant relationship between principal leadership style and the performance of teachers at the college level.

## Literature review

Literature has many shreds of evidence about the leadership and the styles used by various leaders in their organizations for increasing the performance of their workers. While there are various leadership theories and psychology, few of them are more well-known. For example, the behavioral theory of leadership focuses on how leaders behave and assumes that these traits can be copied by other leaders. Sometimes this theory is called the style theory, it suggests that leaders are not born successful, but can be created based on learnable behavior. This theory of leadership is focused heavily on the leader's action.

The second type of leadership theory is contingency leadership, also called situational theory which focuses on the context of a leader. The function of this theory is to look at the situational effects of success or failure. A leader's effectiveness is directly determined by the situational context. While the personality of a leader is a small factor in the success of a leader and the most important factor is the context and situation. This theory suggests that good leaders can adjust their leadership style according to the situation. A contingency theory includes Hershey and Blanchard's Situational Theory, the Evans and House Path-Goal Theory, and Fiedler's Contingency Theory.

Another leadership theory is the great man theory which is also called the trait theory which suggests good leaders are born. They have innate traits and skills that make them great and these are things that cannot be taught or learned. The trait theory suggests that leaders deserve to be in their position because of their special traits.

Among others, management theory is a leadership theory called transactional leadership and focuses on supervision, organization, and group performance of the employees. Transactional leadership is a system of rewards and punishments. Transactional leadership is regularly used in businesses, when employees do something successfully, managers reward them and vice versa. Transactional rewards and punishments are given based on the idea that people only do things for the reward.

Additionally, the following are the leadership styles used by educational leaders: Autocratic, Democratic, and Laissez-Faire. The participative leadership theory is not as common in all types of business. It's called democratic leadership and suggests that employees be directly involved in decision-making in their organization. The leader simply facilitates a conversation and then takes all the suggestions and comes up with the best possible action. In this theory, everyone is very involved with decisions for the team and organization with the leader simply helping direct the charge. Autocratic styles of leadership are known for being authoritative and having the most power in the decision-making process. These types of leaders are known for employing an autocratic leadership style in their leadership style in organization. This type of leader merely gives instructions to group members on how to execute tasks in a given manner, and they avoid establishing obvious lines of communication between employees and followers. Furthermore, these executives never enable employees or other types of workers to participate in the development of organizational policies (Smylie and Jack, 1990; Hoy and Miskel, 1992; John, 2002). Leaders exercising the autocratic style do not ask for any suggestions or initiative from followers. Because it provides great motivation to the leaders, the autocratic leadership style has been successful. It allows for speedy decision-making because only one person makes decisions for the entire group and keeps each conclusion to themselves until he or she deems it is necessary to share it with the rest of the group (Lewin et al., 1939).

Democratic leadership style refers to a situation in which the leader and followers are doing the same amount of labor. This style consists of the leader sharing the decision-making abilities with followers through promoting the interest of the followers and by practicing social equality. Participative leadership or shared leadership are terms used to describe this type of leadership. Any business, including educational institutions, can benefit from this sort of leadership. This approach emphasizes the importance of all members of the group participating in the decision-making process (Research Gate, 2018).

A laissez-faire leadership styles give complete rights and powers to their followers to make decisions to establish goals and work out the problems and hurdles. In this style, decision-making is passed on to the followers. This style focuses on no interference in the affairs of others (Research Gate, 2018). When a leader is hands-off and allows followers to make decisions, this is known as the Laissez-Faire style of administration. Independences are entirely indestructible in Laissez-faire due to group objectives, processes, and operational techniques. These administrators don't intervene too often. This style was identified by Hackman and Johnson (2009) as having the most realistic style, especially when employees are mature and enthusiastic about their work. The laissez-faire leadership style allows for complete autonomy in group decision-making without the involvement of the leaders.

The directive leadership style of principle is similar to the assignment-based method, in which the leader provides teachers with specific rules, standards, and directions for organizing, sorting, as well as completing tasks. When the subordinates' capacity is low and the task at hand is mind-boggling or ambiguous, these techniques are thought to be appropriate. When the boss delivers more directions, the workers are more satisfied (Hoy and Miskel, 2001).

Leaders (Principals) who practice a supportive style of leadership are known for their relationship-oriented style of leadership, which includes the leaders' camaraderie and availability to all employees in the business. This type of leader is continuously concerned about the challenges and concerns of their employees and coworkers. These leaders create a friendly environment for their subordinates and work to improve their employees' lives. It's a powerful technique for assistants who require self-assurance, who want to chip away at unpleasant or upsetting duties, or who don't feel fulfilled at work (Hoy and Miskel, 2001).

Leaders (Principals) that use a consultative style of leadership in their administration have a lot of confidence. These leaders are generous, but they lack confidence and optimism in their employees. Most of the time, these leaders make their final decisions on their own, but they do include their subordinates and seek their input on the problem before establishing any policy inside the college. Workers have positive attitudes toward their employers, administrations, and their jobs. When the workers believe that enough interviews have not been conducted, they freely accept the chief's commands, but they occasionally secretly oppose the request through opposition, particularly when the director decides on a bigger part of the runs guideline (Owens, 1981). The top of the organization is in charge. The majority of the time, center administration assigns tasks to lower-level employees to keep them under control. Examining, evaluating, and administering are all completed. Control is viewed by subordinates as a means of maintaining a high standard (Ukeje, 1998).

Leaders (Principals) who use an achievement-oriented approach to administration are very intelligent, and they present their employees with a variety of challenges that they can meet. These principles involve teachers in achieving the organization's goals and objectives and give rewards for the successful completion of a task. These leaders have strong directive and supporting personalities. They help their followers with their problems and help them in discovering a solution. When achievement-oriented employees are available in the organization, this approach becomes very successful. The administrator who incorporates this method into their leadership style will be able to effortlessly attain their specified goals and objectives (Lussier and Achua, 2001).

Leaders' way of communication and manners with their followers (teachers) are extremely important for the success of any educational institution. According to Oxford (2005), communication is a specific technique for sending any type of information from one person to another. According to Hannagan (2002), communication is a process of passing on information about the feasibility of specific work methods, and it is regarded to take on several aspects. It can be mandated, for example, by defining certain practices that must be completed; motivational, in that it encourages more significant exertion; and mistake-correcting, in that it provides information about the degree of blunder being completed. Whatever the case may be, the importance of communication in educational institutions has been undervalued for a long time, particularly at colleges. Hannagan (2002) went on to say that if we want to achieve a greater level of performance, we can do it with the support of improved and proper communication.

The involvement of the followers (teachers) in the decision-making process by its leader (principal) can boost confidence and improves performance. According to Okumbe (1998), the principal and teachers collaborate on a specific topic or problem and explore strategies to regulate the organization's functioning to enhance the involvement of teachers in the decision-making. Involvement in decision-making is a common occurrence for people who take initiative, but lack of insertion in leadership is associated with autocratic administrations, open innovation is represented, and leaders may hesitantly incorporate personnel in decision-making.

According to The United Nations Educational, Scientific and Cultural Organization (UNESCO), teachers' performance is negatively impacted by their lack of engagement in decision-making processes. “There are significant sensations of remoteness from regional and national-level judgments that are finally joined to educators as unchallengeable decisions, frequently separated as of their everyday state” (UNESCO, 2006). Educators lose confidence in their ability to achieve good and even particular sense alienated and more incompetent in their personalities due to a lack of adequate interactions or conversations. On the other hand, Ndu and Anagbogu (2007) claimed that if educators are not connected in administration, they become outsiders in the college setting. As a result, most professors do not give it their all to have a complete sense of responsibility and loyalty to the college.

Yasmin et al. (2019) found that the impact of transactional and transformational guidance styles has been contrary to teachers' performance. They argued that both leadership styles, for example, transformational and transactional styles of principles of the schools/colleges have not supported improvement in teacher's performance in the short-run.

Saleem et al. (2020) found that the directive leadership style had a significant effect on teacher job performance in the studied schools, followed by the supportive and achievement-oriented leadership styles. Contrariwise, though participative leadership was identified as a significant predictor, it was not considered a favorable predictor of teacher job performance.

Lee et al. (2019) found a link between transformational (but not transactional) leadership and higher levels of supervisory coaching and performance feedback, and that these job resources mediate the relationship between transformational leadership and work engagement. Moreover, the results showed that work engagement mediates the relationships of both supervisory coaching and performance feedback to turnover intention. Generally, Asian leaders can effectively facilitate some aspects of HRD through development-focused behaviors which serve as job resources to boost work engagement and reduce turnover intention.

Teachers' performance improves as principals assign equitably varied responsibilities to teachers. According to Oxford (2005) delegation is the process of delegating rights, power, and responsibility to subordinates. According to Webster (2002) the procedure of cooperating with authorities to accomplish another. Similarly, Okumu (2006) discovered that compelling assignments affect teachers' performance. The findings were fascinating and instructive, they did not reveal how the assignment of responsibilities can improve educator performance at all colleges. Healthfield (2004) examined that for an assignment to be fruitful, the principal must establish designated destinations, determine expert errands, and select who will do them. Chapman (2005) discovered that leaders' involving their followers in the decision-making process, and the equal delegation of duties improved followers' performance. McNamara (2010) stated that for the assigned task to be completed successfully, the boss and the subordinate must agree on when the job is to be completed or if it is a continuing obligation when the survey dates are when the reports are due, and if the project is unpredictable, what assistance the manager can provide. McNamara (2010) found leaders' assigning their subordinates to responsible tasks improved subordinates' performance.

## Data and methodology

The nature of the study is descriptive and data was collected using a survey method through a questionnaire. According to Creswell (2008), descriptive research is the process of examining multiple steps such as quantitative data collecting and analysis to increase prior knowledge about a certain problem or subject. The study's population consisted of all teachers employed in Faisalabad District Government Degree Colleges. A sample of 300 teachers was selected from the sampled colleges in the Faisalabad Division using a simple random selection technique. The data was collected using a self-administered questionnaire on three leadership styles (Autocratic, Democratic, and Laissez-faire) which comprised 30 items about leadership styles and teacher performance^1^. The tool's reliability was calculated to be 0.79. A five-point Likert scale was used in the questionnaire. SPSS was used to analyze the data collected from the respondents (version-20). To explore the differences in the demographic of teachers' perspectives, descriptive and inferential statistics were used (Table 1).

**Table 1:** Demographic statistics of all respondents.

**Items**	**Percentage**
**Gender**
Male	68%
Female	32%
**Locality**
Urban	53%
Rural	47%
**Qualification**
BS	44%
MA/MSc	27%
M.Phil.	19%
Ph.D.	10%
**Professional qualification**
B.Ed	69%
M.Ed	22%
Other	11%
**Teaching subjects**
Arts	60%
Science	40%
**Teaching experience**
1–10 year	55%
11–20 year	36%
Above 20 years	9%

## Data analysis

The results demonstrated in Table 1 shows the demographics statistics of the responded. The demographic variables includes: gender, locality, qualification, professional qualification, teaching subjects and their teaching experience. The results shows that most of our responded in this study are male, living in the urban areas and having BS qualifications. As the professional quantification is compulsory for the teaching, our most of the responded have done B.Ed to fulfill the requirement. As for as our most of the college teachers are teaching arts subjects which are common in the multiple classes and they have teaching experience up to 10 years. Results demonstrated in Table 2 indicate that majority of the college teachers agreed that their principals use a democratic leadership style at a higher level (Mean = 4.52, SD = 1.212), and Laissez-faire at a moderate level (Mean = 3.55, SD = 1.053), and autocratic at a low level (Mean = 3.16, SD = 1.013). Based on said outcomes, this study has discovered that the majority of college principals practice a democratic style of leadership in their colleges. The below graphs also shows the leadership styles practiced by college principals in their colleges.

**Table 2:** Overall mean scores and standard deviation of college teachers' responses toward leadership style used by college principals (*N* = 300).

**S. no**.	**Leadership style**	**Teachers' responses**		**Rank**
		**Mean**	**SDs**	
1.	Autocratic	3.16	1.013	3
2.	Democratic	4.52	1.212	1
3.	Laissez-faire	3.55	1.053	2

A Pearson R correlation test was used to investigate the relationship between principals' leadership style and teachers' performance at the college level.

The results of Table 3 discovered that principals' leadership style had a positive relationship with teacher performance (r = 0.209). The result of *p*-value occurred as (*p* = 0.000 < 0.000 and 0.05 levels). Based on said outcomes, it has been discovered that there had a significant and strong positive relationship between the independent variable (Leadership styles) and the dependent variable (Performance of the teachers). As a result, it has been discovered that when principals increase the use of a democratic leadership style, teacher performance may improve as well. Furthermore, when the principal includes teachers in the decision-making process, communicates well with them, and distributes work evenly, teachers' performance may also improve.

**Table 3:** Relationship with Principals' leadership style and teachers' performance.

**Correlations**					
	**ACL**	**DCL**	**LFL**	**TP**	**Leadership** **style**
ACL (autocratic leadership)	1				
DCL (democratic leadership)	0.086	1			
	0.136				
LFL (lazier-faire leadership)	0.208**	0.162**	1		
	0.000	0.005			
Teacher performance (TP)	0.142*	0.145*	0.131*	1	
	0.014	0.012	0.024		
Leadership style (overall)	0.552**	0.786**	0.592**	0.209**	1
	0.000	0.000	0.000	0.000	

^**^Correlation is significant at the 0.05 level (2-tailed). This symbol shows the significant values in the results. ACL, autocratic leadership; DCL, democratic leadership; LFL, lazier-faire leadership; TP, teacher performance; leadership style (overall).

## Discussion and summary

This study has discovered that the majority of college principals practice a democratic style of leadership in their colleges. The research discovered a strong positive relationship between college principals' leadership styles and teacher performance. The majority of the teachers agreed that democratic leadership is used by principals in their administration. Teachers' performance improves when principals involve them in decision-making, courteously communicate with them, and properly delegate their responsibilities (Tables 2, 3).

College administration is crucial because colleges give education, which is critical for the country builders' financial, sociological, and ethical development. Any college with a bad administration will have a bad education, which will lead to the country's backwardness. Education is deemed to be important for the development of a country, as Panda (2001) has correctly stated that education empowers a country to achieve growth and respect for its citizens. This study's findings are consistent with Nanson's (2010) findings, which found that the democratic leadership style of college administrators has a positive impact on teachers' performance. These findings, as summarized by Okumu (2006) and Nanson (2010), backed up the findings of this study, which found a positive association between college principals' leadership style and teacher performance. The findings of this study are also in line with the findings of Imhangbe et al. (2019) as they also discovered that democratic leadership style had a positive relationship with teachers' job performance. The results of our study also show that the principal democratic leadership style was the most frequent practice of leadership as perceived by the teachers, followed by the autocratic leadership style at the college level. Therefore, both styles of leadership exerted a statistically significant effect on the performance of teachers in college. Unsurprisingly, the practice of these two principals' leadership styles was found to be positive at the college level. On the other hand, the laissez-fair leadership style of the college principal was identified as unhelpful to the performance of teachers. Henceforth, the leadership style of college principals should be autocratic and/or democratic leadership styles in colleges to optimize the performance of teachers. Additionally, the findings of the study also suggest that college principals should encourage teachers to participate in their administration and decision-making.

## Conclusion

This study has discovered that the majority of college principals practice a democratic style of leadership at a higher level, Laissez-faire at a moderate level, and autocratic at a low level in their colleges. Moreover, it has been also discovered that when principals increase the use of a democratic leadership style, teacher performance may improve as well. Furthermore, when the principal includes teachers in the decision-making process, communicates well with them, and distributes work evenly, teachers' performance may also improve. This study recommends that principals working in government colleges should increase their practice of the autocratic style of leadership for enhancing the performance of the teachers at the maximum level.

## Limitations and study forward

This research study is limited to investigating the three leadership styles, autocratic, democratic, and laissez-faire practiced by the Principles in Pakistani colleges located in Punjab only. A comprehensive research study can be conducted by exploring other leadership styles in other provinces of Pakistan.

## Data availability statement

The raw data supporting the conclusions of this article will be made available by the authors, without undue reservation.

## Ethics statement

The studies involving human participants were reviewed and approved by Shaanxi Normal University, Xi'an, China. The patients/participants provided their written informed consent to participate in this study.

## Author contributions

US: conceptualization, data curation, validation, and writing—original draft. QY: funding acquisition. RT: investigation and resources. US and QY: methodology. US and RT: writing—review and editing. All authors contributed to the article and approved the submitted version.

## Conflict of interest

The authors declare that the research was conducted in the absence of any commercial or financial relationships that could be construed as a potential conflict of interest. The handling editor MA declared a shared affiliation with the author RT at the time of review.

## Publisher's note

All claims expressed in this article are solely those of the authors and do not necessarily represent those of their affiliated organizations, or those of the publisher, the editors and the reviewers. Any product that may be evaluated in this article, or claim that may be made by its manufacturer, is not guaranteed or endorsed by the publisher.

## Footnotes

^1^See Appendix-I.

## References

[B38] Abbas A. Saud M. Usman I. Ekowati D. (2020). Servant leadership and religiosity: An indicator of employee performance in the education sector. Int. J. Innov. Creativity Change 13, 391–409.

[B1] Bhoomireddy N. (2004). School Organization, Management, and Administration. Ludhiana: Kalyani Publishers.

[B2] Chapman A. (2005). Effective Delegation Skills, Delegation Techniques, Process. Available online at: https://gnpublication.org (accessed October 10, 2018).

[B3] Chin R. (2015). Examining teamwork and leadership in the fields of public administration, leadership, and management. Team Perform. Manage. 21, 199–216. 10.1108/TPM-07-2014-0037

[B4] Creswell J. S. (2008). Researcher methods in educational environments. Educ. Action Res. 10, 233–251. 10.15700/saje.v28n3a176

[B5] Crum K. S. Sherman W. H. (2008). Facilitating high achievement: high school principals reflections on their successful leadership practices. J. Educ. Adm. 46, 562–580. 10.1108/09578230810895492

[B6] Department of the Army (2006). Army Leadership. Competent, Confident, and Agile. FM 6-22. Washington, D.C.: Department of the Army.

[B7] Goel O.P. (2005). School Organization and Management. Adarash Nagar: Isha Books, 170–173.

[B8] Hackman P. Johnson C. E. (2009). Leadership: A Communication Perspective, 5th Edn. Long Grove, IL: Waveland Press.

[B9] Hannagan T. (2002). Management: Concepts and Practice. London: Pitman Publishing Pearson Education Limited.

[B10] Healthfield S. M. (2004). Delegation as a Leadership Style: Tips for Effective Delegation.

[B11] Hoy N. K. Miskel C. G. (1992). Educational Administration: Theory, Research and Practice. 2nd Edn. New York: Randam House, 22–24.

[B39] Hoy W. K. Miskel C. G. (2001). Educational Administration, Theory, Research and Practices. 6th Edn, New York, NY: Mcgraw Hall.

[B12] Imhangbe O. Okecha R. Obozuwa J. (2019). Principals' leadership styles and teachers' job performance: evidence from Edo State, Nigeria. Educ. Manage. Adm. Leadership 47, 909–924. 10.1177/1741143218764178

[B13] John C.M. (2002). Million Leaders Mandate. Notebook One. Equip Publishers.

[B14] Lee M. C. C. Idris M. A. Tuckey M. (2019). Supervisory coaching and performance feedback as mediators of the relationships between leadership styles, work engagement, and turnover intention. Hum. Resource Dev. Int. 22, 257–282. 10.1080/13678868.2018.1530170

[B15] Lewin K. Lippitt R. White R. K. (1939). Patterns of aggressive behavior in experimentally created social climates. J. Soc. Psychol. 10, 271–301. 10.1080/00224545.1939.9713366

[B16] Lussier R. N. Achua C. F. (2001). Leadership: Theory, Application and Skill Development. South- West College Publishing.

[B17] Maqbool S. Ismail S. Maqbool S. Zubair M. (2019). Principals' behavior and teachers' performance at secondary schools in rural area of Pakistan. Int. J. Acad. Res. Bus. Soc. Sci. 9, 788–801. 10.6007/IJARBSS/v9-i1/5481

[B18] McNamara C. (2010). Basis of Conflict Management: Field Guide to Leadership and Supervision.

[B19] Nanson P.K. (2010). Leadership Styles and Teachers' Performance in Secondary School in Nakaseke District. (Unpublished MA Thesis), Kampala: Makerer University.

[B20] Ndu A.A. Anagbogu M.A. (2007). Framework for Effective Management of University's in the 21st Century in Issues in Higher Education: Research-Evidence From Sub-Saharan Africa.

[B21] Northouse P. G. (2018). Leadership: Theory and Practice, 8 Edn. Thousand Oaks, CA: Sage Publication.

[B22] Okumbe J.A. (1998). Educational Management Theory, a Comparative Evolution to General Theory. Nairobi: Nairobi University Printery.

[B23] Okumu F.M. (2006). An investigation Into Delegation and Its Effects on Management of Secondary Schools in Kampala District, Uganda. (Unpublished Masters (Educ. Mgt) dissertation), Makerer e University, Kampala, Uganda.

[B24] Owens R. G. (1981). Organizational Behavior in Education. 2nd Edn. Englewood Cliffs: Prentice Hall.

[B25] Oxford (2005). Advanced Learner's Dictionary. Oxford: OxfordUniversity Press.

[B26] Panda U.N. (2001). School Management. New Dehli: Ashish Publishing House, 2–3.

[B27] Phuc T. Q. B. Parveen K. Tran D. T. T. Nguyen D. T. A. (2021). The linkage between ethical leadership and lecturer job satisfaction at a private higher education institution in Vietnam. J. Soc. Sci. Adv. 2, 39–50. 10.52223/JSSA21-020202-12

[B28] Quraishi U. Aziz F. (2018). An investigation of authentic leadership and teachers' organizational citizenship behavior in secondary schools of Pakistan. Cogent Educ. 5, 1437670. 10.1080/2331186X.2018.1437670

[B29] Research Gate (2018). Modern Leadership and Management Methods for Development Organizations. Research Gate.

[B30] Saleem A. Aslam S. Yin H. B. Rao C. (2020). Principal leadership styles and teacher job performance: viewpoint of middle management. Sustainability 12, 3390. 10.3390/su12083390

[B31] Smylie M. A. Jack W. D. (1990). Teachers Leadership Tension and Ambiguities in Organizational Perspective. Educ. Admin., 26–59.

[B32] Sungtong E. (2007). Leadership Challenges to Public Secondary School Principals in the Era of Education Reform and Culture Unrest in Boarder Provenance of Southern Thailand. Faculty of the Graduate School at the University of Missouri Columbia, USA

[B33] Ukeje B. (1998). Educational Administration. Enugu: Fourth Dimension Publishing Co. Ltds.

[B40] UNESCO (2006). Policy Breife, School leadership in central Asia. Available online at: https://unesdoc.unesco.org/ark:/48223/pf0000370957

[B34] Webster (2002). International Dictionary. Massachusetts: Mariam Webster Inc.

[B35] Western S. (2013). Leadership: A Critical Text. London: Sage.

[B36] Wu H. Shen J. Zhang Y. Zheng Y. (2020). Examining the effect of principal leadership on student science achievement. Int. J. Sci. Educ. 42, 1017–1039. 10.1080/09500693.2020.1747664

[B37] Yasmin F. Imran M. Sultana M. (2019). Effects of principals' leadership styles on teachers' performance at secondary schools in dera ismail khan. Glob. Soc. Sci. Rev. 4, 281–286. 10.31703/gssr.2019(IV-I)0.37

