# Supplementary material for: Principals' leadership styles and its impact on teachers' performance at college level
Source: Front Psychol. 2022 Sep 6;13:919693. doi: 10.3389/fpsyg.2022.919693 (PMC9518751; doi:10.3389/fpsyg.2022.919693)
Supplement: Supplementary file 1 [file Data_Sheet_1.docx]

**Questionnaire**

Respected Teachers,

I am a Ph.D. scholar and writing a research article. In this regard, your opinion is required. I hope you will take a few minutes to complete this questionnaire as your views will help to improve principals' leadership styles. Our information will be kept confidential and the results will be only for research purposes.

Uzma Sarwar

School of Education Shaanxi Normal University Xi’an, 710062 RP China.

Email: uzmasarwar15@gmail.com

| **Demographics** | | | | | |
| --- | --- | --- | --- | --- | --- |
| 1 | Name(Optional): | | | | |
| 2 | Name of college: (Urban/Rural) | | | | |
| 3 | Gender: | Male |  | Female |  |
| 4 | Teacher of | (Arts) |  | (Science) |  |
| 5 | Qualification | Academic |  | Professional |  |
| 6 | Experience in years: |  | | | |
| 7 | Subject Currently Teaching |  | | | |

Instructions: Please rate the statement on the given 5-point rating scale. Tick (√) the most appropriate one against each statement.

SDA = Strongly Disagree, DA = Dis Agree, ND = Not decided, SA = Strongly Agree, A = Agree

| **Sr#** | **Autocratic Style** | **SDA** | **DA** | **ND** | **SA** | **A** |
| --- | --- | --- | --- | --- | --- | --- |
| 1 | My principal considers me as subordinate |  |  |  |  |  |
| 2 | My principal never involve me in the decision making process |  |  |  |  |  |
| 3 | My principal uses hard rules and regulations in college |  |  |  |  |  |
| 4 | My principal discouraged teachers' suggestions in college matters |  |  |  |  |  |
| 5 | My principal implement their own ideas in college |  |  |  |  |  |
|  | **Democratic Style** |  |  |  |  |  |
| 6 | My principal considers me as a team member |  |  |  |  |  |
| 7 | My principal involve me in decision-making process |  |  |  |  |  |
| 8 | My principal provide me guidance without any pressure |  |  |  |  |  |
| 9 | My principal showed supportive communication with me |  |  |  |  |  |
| 10 | My principal help teachers find their "passion" |  |  |  |  |  |
|  | **Laissez-Faire Style** |  |  |  |  |  |
| 11 | My principal order to solve the problem at my ends |  |  |  |  |  |
| 12 | My principal never appraise my work/performance |  |  |  |  |  |
| 13 | My principal never give me the freedom to solve the problem at my ends |  |  |  |  |  |
| 14 | My principal provide little input towards my problems |  |  |  |  |  |
| 15 | Generally, my principal leave me alone to complete my job and make decision |  |  |  |  |  |
|  | **Teachers’ Performance** |  |  |  |  |  |
| 16 | The autocratic leadership style of the principal improved my performance |  |  |  |  |  |
| 17 | The democratic leadership style of the principal enhanced my performance |  |  |  |  |  |
| 18 | The Laissez-faire leadership style of the principal increased my performance |  |  |  |  |  |
| 19 | My problem-solving skills have been improved |  |  |  |  |  |
| 20 | My communication skills have been improved |  |  |  |  |  |
| 21 | My teaching skills have been increased |  |  |  |  |  |
| 22 | My analytical skills have been developed |  |  |  |  |  |
| 23 | My motivational abilities have been improved |  |  |  |  |  |
| 24 | My negotiation skills have been improved |  |  |  |  |  |
| 25 | My research skills have been improved |  |  |  |  |  |
| 26 | My lesson planning skills have been improved |  |  |  |  |  |
| 27 | My lesson delivery skills have been increased |  |  |  |  |  |
| 28 | I can use various aids in my teaching learning |  |  |  |  |  |
| 29 | The principal's encouragements increased my performance |  |  |  |  |  |
| 30 | Principal’s appreciation enhanced my performance |  |  |  |  |  |

Any Other Remarks: __________________________________________________________

****Thank You****
